# lncRNA-PACER upregulates *COX-2* and PGE2 through the NF-κB pathway to promote the proliferation and invasion of colorectal-cancer cells

**DOI:** 10.1093/gastro/goaa060

**Published:** 2020-12-10

**Authors:** Peng Sun, Ji-Chuan Quan, Song Wang, Meng Zhuang, Zheng Liu, Xu Guan, Gui-Yu Wang, Hong-Ying Wang, Xi-Shan Wang

**Affiliations:** 1 Department of Colorectal Surgery, The Second Affiliated Hospital of Harbin Medical University, Harbin, Heilongjiang, P. R. China; 2 Department of Gastrointestinal Surgery, Shenzhen Hospital, National Cancer Center/Cancer Hospital, Chinese Academy of Medical Sciences and Peking Union Medical College, Shenzhen, Guangdong, P. R. China; 3 Department of Colorectal Surgery, National Cancer Center/Cancer Hospital, Chinese Academy of Medical Sciences and Peking Union Medical College, Beijing, P. R. China; 4 Department of State Key Laboratory of Molecular Oncology, National Cancer Center/Cancer Hospital, Chinese Academy of Medical Sciences and Peking Union Medical College, Beijing, P. R. China

**Keywords:** p50-associated cyclooxygenase-2 extragenic RNA (PACER), colorectal cancer (CRC), lncRNA, cyclooxygenase-2 (COX-2)

## Abstract

**Background:**

p50-associated cyclooxygenase-2 extragenic RNA (PACER) is a recently identified antisense long non-coding RNA (lncRNA) located on the upstream of the promoter region of cyclooxygenase-2 (*COX-2*). Preliminary studies have suggested that PACER is involved in the regulation of *COX-2* expression in macrophagocyte and osteosarcoma cells. However, the role of this lncRNA in colorectal cancer (CRC) remains elusive. Here, we investigated the expression of PACER and its effect on cell proliferation and invasion to explore the role of PACER in CRC.

**Methods:**

Real-time quantitative PCR (RT-qPCR) analysis was used to evaluate the expression of PACER in CRC tissues and cells. Methyl thiazolyl tetrazolium (MTT) analysis was then used to investigate the inhibition effect of PACER knock-down in cell proliferation. The promoting role of this lncRNA on invasion by CRC cells was analysed by wound-healing assays, colony-formation assay, and transwell assays. We then used fluorescence *in situ* hybridization (FISH) to establish the subcellular localization of PACER. COX-2 protein levels were quantified by Western blot analysis and grayscale scanning analysis following the knock-down of PACER. Luciferase assay was carried out to monitor the modulation of the COX-2 promoter region by PACER. Tumor xenografts models were used to investigate the impact of PACER on the tumorigenesis of CRC cells *in vivo*. Enzyme-linked immunosorbent assay (ELISA) was then used to quantify prostaglandin E2 (PGE2) production upon knock-down of PACER.

**Results:**

RT-qPCR analysis revealed that PACER was highly expressed in CRC tissues and cells, and a high PACER-expression level was associated with poor prognosis. MTT assay, wound-healing assay, colony-formation assay, and transwell assay revealed that PACER enhanced CRC-cell proliferation, invasion, and metastasis *in vitro*. Analysis of lncRNA localization by FISH showed that it mainly resided in the nucleus. RT-qPCR showed that PACER increased mRNA levels of *COX-2*. Western blot analysis demonstrated, under normal circumstances, that knock-down of PACER decreased the COX-2 protein level. In the case of p50 absence, COX-2 protein increased rapidly and remained highly expressed after knocking down PACER. Luciferase assay revealed that PACER modulated the *COX-2* promoter region. Mouse xenograft models of CRC revealed that PACER promoted colorectal tumorigenesis *in vivo*. ELISA revealed that PACER knock-down inhibited PGE2 production.

**Conclusions:**

PACER modulates *COX-2* expression through the nuclear factor kappa B (NF-κB) pathway in CRC. An increased level of PACER enhances proliferation, migration, and invasion of tumor cells by increasing COX-2 and PGE2 synthesis.

## Introduction

Colorectal cancer (CRC) is one of the most prevalent malignant tumors globally [1]. It is estimated that CRC affects >1 million people annually and the disease-specific mortality rate in developed countries is nearly 33% [2, 3]. Its pathogenesis and progression involve a complex interplay of multiple genomic and epigenetic changes [4, 5]. Recent studies have examined the role of various genes in the pathogenesis of CRC [6, 7]. In the human genome, protein-coding genes account for only ∼2%. In addition, the other RNAs lack protein-coding ability or only encode small peptide chains, and are thus known as non-protein-coding RNAs [8]. These non-protein-coding RNAs include small interfering RNA (siRNA), microRNA (miRNA), small nucleolar RNA (snoRNA), and long non-coding RNA (lncRNA) [9].

lncRNAs are nuclear or cytoplasmic RNA transcripts consisting of >200 nucleotides in length [10]. With >23,000 lncRNAs identified to date, it is estimated that the number of lncRNAs may greatly surpass that of protein-coding genes [11]. Multiple studies have established that lncRNAs modulate various cellular processes including chromosome modification, transcription, and post-transcriptional modification [12]. Several lncRNAs showed aberrant expression in CRC. For instance, MALAT1, CCAT2, CCAT1-L, and HOTAIR all showed upregulation [13–15], whereas the expression of GAS5 and MEG3 was downregulated [16, 17]. Consequently, dysregulation of these lncRNAs often leads to altered function of their target genes that, in turn, may accelerate CRC progression [18, 19].

Cyclooxygenase 2 (*COX-2*) is the key pro-inflammatory enzyme that catalyses arachidonic acid into prostaglandins, which is involved in inflammatory diseases and certain types of tumor [20]. *COX-2* and its products prostaglandins, especially prostaglandin E2 (PGE2), are important mediators of various biological processes, including inflammation, fever, and tumorigenesis [21, 22]. Intriguingly, recent studies have suggested that *COX-2* is frequently aberrantly expressed in various cancers and promotes tumorigenesis in breast cancer, prostate cancer, lung cancer, and especially in CRC [23, 24]. A previous study reported that bromodomain and plant homeodomain finger transcription factor (BPTF) could function as a transcriptional regulator of the nuclear factor kappa B (NF-κB) pathway and increased *COX-2* transcription in lung cancer, which eventually leads to poor prognosis [25]. Another study on triple-negative breast cancer showed that COX-2 protein was upregulated because the ubiquitination and proteasomal degradation were inhibited by centromere protein U (CENPU; a centromere component essential for mitosis) [26]. Recently, Wang *et al*. revealed that stromal interaction molecule 1 (STIM1) may promote CRC-cell migration through increasing the expression of COX-2 and production of PGE2, while the depletion of STIM1 with short-hairpin RNA inhibited CRC-cell migration [27].

The p50-associated *COX-2* extragenic RNA (PACER) is a recently identified lncRNA that modulates *COX-2* gene expression in primary human mammary cells and macrophagocytes [28]. PACER locates on chromosome 1 and is transcribed from the sequences that are upstream promoter regions of *COX-2*. The objective of the present study is to investigate the role of PACER in CRC. In this study, we uncovered an oncogenic function of PACER during CRC tumorigenesis. The expression of PACER in CRC tissues and cell lines was determined, and the function of PACER on proliferation and invasion was investigated via gene-knock-down experiments *in vivo* and *in vitro*. Furthermore, the mechanisms of regulating the relationship between PACER and *COX-2* in CRC were explored.

## Materials and methods

### Patient samples

Cancer tissues and adjacent tissues were collected from 46 patients who underwent radical resection surgery for CRC as the initial treatment at the Cancer Hospital, Chinese Academy of Medical Sciences and Peking Union Medical College between June 2012 and June 2013. The study protocol was approved by the ethical committee of the Cancer Hospital, Chinese Academy of Medical Sciences. All tumor samples were pathologically diagnosed as adenocarcinomas after operation. All patients were followed up for at least 60 months. Patients who were lost during the follow-up period were excluded from the final analysis. All clinical samples were collected with informed consent from the patients.

### Cell culture

Human SW480, HT29, DLD-1, HCT116, RKO, SW620, and CCD-841 cells (ATCC, Manassas, VA, USA) were cultured in RMPI 1640 medium containing 10% fetal bovine serum (FBS; GIBCO, Brooklyn, NY, USA). All cell lines were cultured in a cell incubator (Thermo Scientific, Asheville, NC, USA) under stable conditions of 5% CO_2_ at 37°C.

### RNA extraction and quantitative PCR

The CRC cells were washed twice with 4°C phosphate-buffered saline (PBS; GIBCO) and treated with Trizol reagent (Thermo Scientific) to extract total RNA. A total of 2 μg RNA was reverse-transcribed to complementary DNA (cDNA) using the RevertAid First Strand cDNA Synthesis Kit (Thermo Fisher Scientific, Waltham, MA, USA) following the manufacturer’s instructions. Subsequently, real-time quantitative PCR (RT-qPCR) was performed in triplicate using 1 μL of cDNA in a total reaction mixture of 20 μL in Bio-rad iCycler PCR System (Bio-Rad, Hercules, CA, USA). The relative mRNA expression was calculated using the 2^–△△Ct^ method, with GAPDH as the internal reference gene of PACER and β-actin as the internal reference gene of *COX-2*. The primers sequences were designed using the PrimerBlast tool in the PubMed database (www.ncbi.nlm.nih.gov/tools/primer-blast/) and synthesized by Sangon Biotech (Shanghai, China). The primer sequences are listed in Table 1.

**Table1.** Primer sequences used in PCR and annealing temperatures

Primer name	Primer sequence	Amplicon length (bp)	Annealing temperature (°C)
PACER	Forward: 5′-CTCCACGGGTCACCAATATAAA-3′ Reverse: 5′-ACGCATCAGGGAGAGAAATG-3′	126	58
GAPDH	Forward: 5′-CTCTGCTCCTCCTGTTCGAC-3′	121	60
	Reverse: 5′-GCGCCCAATACGACCAAATC-3′		
COX-2	Forward: 5′-CTGCGCCTTTTCAAGGATGG-3′	135	60
	Reverse: 5′-CCCCACAGCAAACCGTAGAT-3′		
β-actin	Forward: 5′-TGACAGGATGCAGAAGGAGA-3′	75	58
	Reverse: 5′-CGCTCAGGAGGAGCAATG-3′		

### Oligonucleotide transfection

siRNAs of PACER were designed and customized according to the nucleotide sequence of PACER by Genepharma (Shanghai, China). These siRNAs were transfected into SW480 and DLD-1 cells using Lipofectamine 2000 (Invitrogen, Carlsbad, CA, USA) according to the manufacturer’s protocols. Non-specific siRNA (NS-siRNA) served as control. The sequences of siRNAs were presented as follows: siRNA-1 (sense, 5′-CCA CGG GUC ACC AAU AUA ATT-3′; antisense, 5′-UUA UAU UGG UGA CCC GUG GTT-3′), siRNA-2 (sense, 5′-CAU AGG AGA UAC UGG UAA ATT-3′; antisense, 5′-UUU ACC AGU AUC UCC UAU GTT-3′), and NS-siRNA (sense, 5′-UUC UCC GAA CGU GUC ACG UTT-3′; antisense, 5′-ACG UGA CAC GUU CGG AGA ATT-3′).

### MTT assay

The MTT (methyl thiazolyl tetrazolium) assay was performed to determine the proliferation ability of SW480 and DLD-1 cells after transfection. Two thousand cells were plated in each well of 96-well plates (Coning, New York, NY, USA) and each group was prepared in 5 duplicate wells. In each well, 50 μL of 1 mg/mL MTT solution (Sigma, St Louis, MO, USA) was added on the third day, the fifth day, and the seventh day. After cultivation for 4 h at 37°C, we discarded the supernatant and added 100 μL dimethyl sulfoxide (DMSO; Sigma) to each well. The plate was horizontally shaken for 15 min in a thermostatic oscillator (Leopard Scientific Instruments Co., Beijing, China). Finally, the plate was read using an iMARK microplate reader (Bio-Rad) at 490 nm and the optical density values were analysed.

### Wound-healing assay

To evaluate the migration ability of cells, the wound-healing assay was performed. Transfected cells (4 × 10^5^ per well) were seeded into six-well plates and continuously incubated until a monolayer of cells was evenly spread on the entire bottom of the plate. A scratch wound was created on the cell surface slowly and uniformly using a micropipette tip. The plate was rinsed with PBS several times until all unattached cells were removed; the residual cells were then cultured with 1640 medium without FBS. The wounds were photographed under an inverted microscope (Leica, Wetzlar, Germany) at 0, 24, and 48 h. The images were analysed using ImageJ 1.8.0 software (National Institutes of Health, Bethesda, MD, USA).

### Transwell assay

Cell-invasion ability was evaluated using the transwell assay. The transfected cells were starved for 12 h in serum-free medium prior to experimentation. The cells were rinsed once or twice using PBS, digested, and resuspended in the serum-free 1640 medium at a density of 2 × 10^5^ cells/mL. Next, 200 μL of the cell suspension was added into the upper layer of the transwell chamber (Corning) covered with Matrigel (BD Biosciences, San Jose, CA, USA), while 500 μL of 10% FBS medium was added to the lower chamber. The transwell chamber was cultured for 30 h in a cell incubator. Thereafter, the chamber was removed from the incubator and the cells were fixed with methanol for 30 min. Subsequently, cells were stained with 0.1% crystal violet solution for 20 min and then rinsed several times using deionized water. The cells on the upper surface of the chamber bottom were wiped gently with a cotton swab. The invaded cells on the lower surface were observed and images taken under an inverted microscope (Leica).

### Colony-formation assay

Cells transfected with siRNAs were seeded in 6-well plates at a density of 300 cells per well. The fresh medium containing 10% FBS was replaced every 4 days. The proliferating colonies were stained with crystal violet on the twelfth day. The plates were observed with an imaging system (Syngene GBOX, Cambridge, UK) and images were acquired. The number of colonies formed was counted using ImageJ 1.8.0 software (National Institutes of Health). The rate of colony formation was calculated using the equation: colony-formation rate = (number of colonies/number of cells incubated)  × 100%.

### Establishment of a stable PACER-knock-down cell line and plasmid transfection

The short-hairpin RNA (shRNA) sequence targeted at PACER and scramble sequence (which was non-homologous to any human genome sequences and acted as the negative control) was inserted into the vector (pLent-GFP-Puro), which was synthesized and purchased from Vigene Biosciences (Jinan, Shandong, China). Both the shNC (negative control) and the shPACER plasmids were transfected into SW480 cells using the lentiviral packaged method according to the manufacturer’s protocol. Puromycin was used to select the monoclones with shPACER stable expression, which were then incubated to establish stable PACER-knock-down cell lines.

### LncRNA fluorescence *in situ* hybridization

Fluorescence *in situ* hybridization (FISH) was performed using a RiboTM Fluorescent In Situ Hybridization Kit (RiboBio, Guangzhou, Guangdong, China) and PACER-specific probes. U6 probes and 18S probes were utilized as the nuclear and cytoplasmic controls, respectively. SW480 cells were plated in a laser confocal Petri dish (Jingan Technologies Inc., Shanghai, China). When the cells became 60%–70% confluent, they were fixed in 4% paraformaldehyde for 10 min. Next, 200 μL pre-hybridization solution was added to each well and blocked for 30 min at 37°C. Subsequently, 2 μL lncRNA-PACER FISH Probe or 2 μL internal control FISH Probe Mix was separately mixed with 200 μL hybridization solution in darkness and then hybridized overnight at 37°C. They were subsequently stained using DAPI (4',6-diamidino-2-phenylindole) for 10 min and observed using a laser scanning confocal microscope (Olympus, Tokyo, Japan).

### Luciferase-reporter assay

SW480 cells were seeded in 24-well plates at a density of 2 × 10^5^ cells per well and cultured overnight before transfection. The cells were co-transfected with a mixture of 0.4 μg shPACER plasmid, 0.4 μg XP2-h*COX-2*-LUC reporter plasmid (which was inserted the sequence of *COX-2* promoter region, purchased from Biovector NTCC Inc., Beijing, China) [29, 30], and 0.1 μg Renilla-luciferase reporter vectors (Biovector NTCC Inc.). After 30 h of transfection, 1 μg lipopolysaccharide (LPS; Cell Signaling Technology, Danvers, MA, USA) was added into each well and incubated for 8 h. The luciferase activity was measured using a dual luciferase-reporter-assay system (BioTek, Winooski, VT, USA). For comparison, the relative luciferase activity was normalized to that of Renilla-luciferase activity. Finally, the fold change of fluorescence was calculated in relation to the control group.

### Western blotting

Total proteins were extracted from cell lines with the mixed liquor of RIPA (radioimmunoprecipitation assay) lysis buffer (Beyotime, Shanghai, China), supplemented with 0.01% EDTA (ethylenediaminetetraacetic acid) and 10% proteinase inhibitor. The protein concentration was determined using a BCA protein assay kit (Bio-Rad). The total proteins were separated by 10% SDS-PAGE (sodium dodecyl sulfate polyacrylamide gel electrophoresis) and then transferred to polyvinylidene difluoride membranes. The membranes were blocked by 5% skimmed milk for 2 h, then incubated overnight at 4°C using the primary antibody against human COX-2 (1:1,000; Cell Signaling Technology), β-actin (1:10,000; Abcam, Cambridge, MA, USA), NF-κB1 p50 (1:1,000; Cell Signaling Technology). The membranes were further incubated using horseradish peroxidase anti-rabbit IgG (1:10,000; Cell Signaling Technology) for 1 h. The protein bands were visualized with ECL (enhanced chemiluminescence) liquid (Applygen Technologies Inc., Beijing, China) using Amersham Imager 600 equipment (GE Healthcare, Chicago, IL, USA). β-actin was utilized as the loading control.

### Subcutaneous tumor xenografts models in nude mice

Fourteen 3-week-old male BALB/c nude mice, weighing between 18 and 20 g, were purchased from HFK Bioscience Co. Ltd (Beijing, China) and randomly divided into two groups. The SW480 cells with shPACER or shNC stable transfection were redigested and harvested. Subsequently, 100 μL of PBS containing 2 × 10^6^ cells was injected into the right-side subcutis of nude mice. Xenograft tumor volume was monitored and calculated by measuring the largest (length) and the shortest (width) diameters using digital calipers every 3 days. The tumor volume was calculated using the following formula: volume = length × width^2^ × 0.5. The tumors were excised from the nude mice and weighed after 21 days. The xenograft tumor tissue samples were fixed with 4% neutral formalin, embedded in paraffin, and then sliced for immunohistochemistry analysis.

### Database analysis

To characterize the expression of PACER in CRC patients, we analysed The Cancer Genome Atlas (TCGA) database using Gene Expression Profiling Interactive Analysis (GEPIA) [31] and the Human Cancer Metastasis Database (HCMDB) interactive web application [32].

### Statistical analysis

Data are presented as the means ± SD (standard deviation) and each value represents at least three independent experiments. Error bars in the scatter plots and the column graphs represent SD. The means of different groups were compared by one-way analysis of variance. The survival difference between the two groups was determined using Kaplan–Meier survival curves and log-rank tests. Correlation analysis was performed using Pearson’s correlation test. *P-*value <0.05 was considered statistically significant. Data were analysed by using GraphPad Prism (version 6.01, GraphPad Software, San Diego, CA, USA).

## Results

### PACER upregulation in CRC associates with poor prognosis

**Figure 1.**
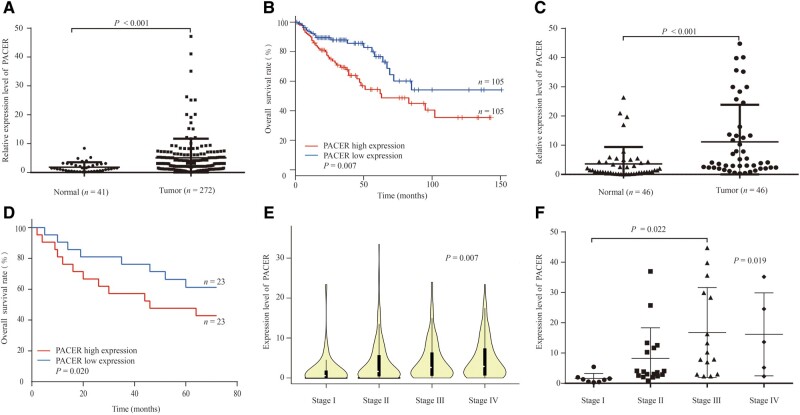
The expression of PACER in CRC-tissue samples. (A) The expression of PACER in 41 normal-tissue samples and 272 CRC-tissue samples from the TCGA database (*P* < 0.001). (B) Kaplan–Meier analyses of overall survival (OS) for PACER expression in the TCGA database (*P* = 0.007). (C) The expression of PACER in 46 pairs of normal-tissue and cancer-tissue samples was detected by RT-qPCR assays (*P* < 0.001). (D) The overall survival (OS) for PACER expression in CRC samples (*P* = 0.020). (E) Increased PACER expression in tumor tissue was associated with TNM stages based on data from the TCGA database (*P* = 0.007). (F) The average expression of PACER increased with TNM stages in 46 patients (*P* = 0.019). PACER, p50-associated cyclooxygenase-2 extragenic RNA; CRC, colorectal cancer.

Based on TCGA data, our analyses revealed that PACER expression is significantly upregulated in CRC tumors (*P* < 0.001; Figure 1A) and upregulation of PACER expression is associated with poor CRC prognosis (*P* = 0.007; Figure 1B). We then evaluated the expression of PACER in 46 paired human CRC tissues and found that the expression of PACER in cancer tissues is higher than that in adjacent normal tissues (*P* < 0.001; Figure 1C). Analysis of CRC survival using the Kaplan–Meier strategy demonstrated that patients whose tumors exhibited high PACER expression have a significantly worse prognosis (*P* = 0.020; Figure 1D). Further analysis of the TCGA database as well as our CRC samples indicated that high average expression levels of PACER are associated with CRC TNM staging (Figure 1E and F). However, the small sample size for the stage analysis limits the significance of the results; the high average expression of PACER is only significantly different between stages I and III (Figure 1F). Taken together, these results showed that PACER is frequently upregulated in CRC patients and that higher expression levels are associated with poor prognosis.

### PACER promotes cell proliferation and invasion *in vitro*

**Figure 2.**
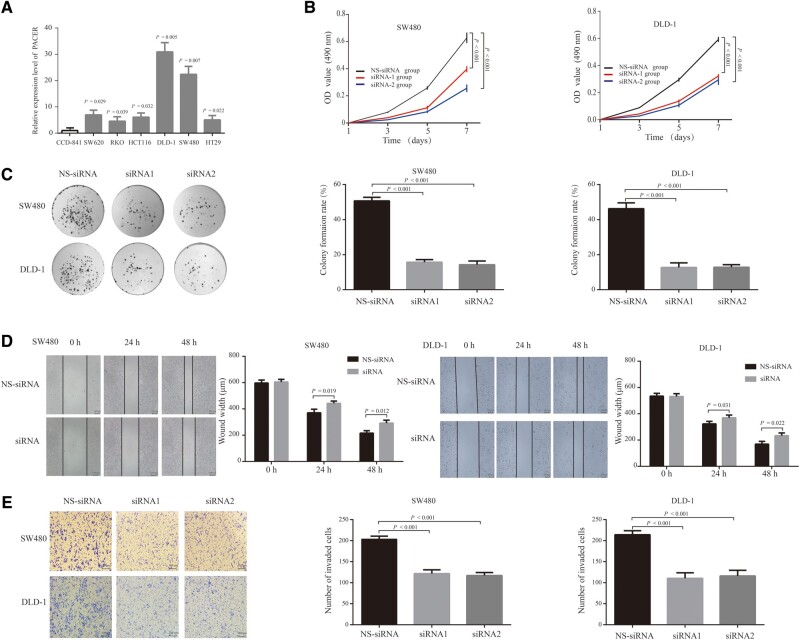
PACER knock-down negatively regulated the proliferation and migration of CRC cells. (A) Using CCD-841 as the control group, the fold change of CRC-cell lines in relation to CCD-841 cells was determined. (B) MTT assay results of the NS-siRNA group and siRNA group in SW480 cells and DLD-1 cells (*P* < 0.001). (C) Cells transfected with siRNAs were seeded in six-well plates and cultivated for 12 days to determine the colony-formation rate (*P* < 0.001). (D) The width of the wound in the SW480 group and DLD-1 group at 0, 24, and 48 h. (E) In the transwell assay, cells were serum-starved for 8 h before seeding on the upper transwell chamber. After 30 h of cultivation, cells that invaded the lower chamber were counted (*P* < 0.001). All data are shown as means ± SD (*n* = 3). PACER, p50-associated cyclooxygenase-2 extragenic RNA; CRC, colorectal cancer; NS-siRNA, non-specific small interfering RNA.

The expression analysis revealed that PACER is highly expressed in the CRC-cell lines SW620 (*P* = 0.029), SW480 (*P* = 0.007), DLD-1 (*P* = 0.005), RKO (*P* = 0.039), HT29 (*P* = 0.022), and HCT116 (*P* = 0.032) relative to CCD-841 cells (Figure 2A). We elected to use the CRC-cell lines SW480 and DLD-1 for downstream experiments as they exhibited high proliferative and invasive capability. To evaluate the effect of PACER on the proliferation of CRC cells, we used two siRNAs (siRNA-1 and siRNA-2) to knock down PACER in SW480 and DLD-1 cell lines. Analysis of PACER expression showed that the two oligos potently inhibited its expression relative to non-specific knock-down cells (Supplementary Figure 1). In order to establish the role of PACER in CRC-cell proliferation, we carried out an MTT assay and observed reduced proliferation rates upon PACER knock-down relative to the non-specific knock-down group (all *P* < 0.001; Figure 2B). To investigate the effect of this lncRNA on CRC oncogenesis, we performed colony-formation assays and observed that, relative to non-specific knock-down cells, fewer colonies were formed in PACER-knock-down CRC cells than in non-specific knock-down cells (all *P* < 0.001; Figure 2C). Taken together, these data suggested that PACER may promote CRC oncogenesis by modulating the proliferation and colony-forming capacity of CRC cells. To investigate the function of PACER in CRC-cell migration and invasion, we carried out wound-healing assays and observed that, relative to non-specific knock-down cells, CRC migration was impaired by PACER knock-down (Figure 2D), although the phenomena were not as evident as proliferation. Transwell-invasion assays revealed that PACER knock-down strongly reduced CRC-cell-invasive potential (all *P* < 0.001; Figure 2E). Collectively, these observations indicate that PACER promotes the proliferation and invasion of the CRC cells, and may therefore modulate CRC malignancy.

### PACER regulates *COX-2* through the NF-κB-signaling pathway

**Figure 3.**
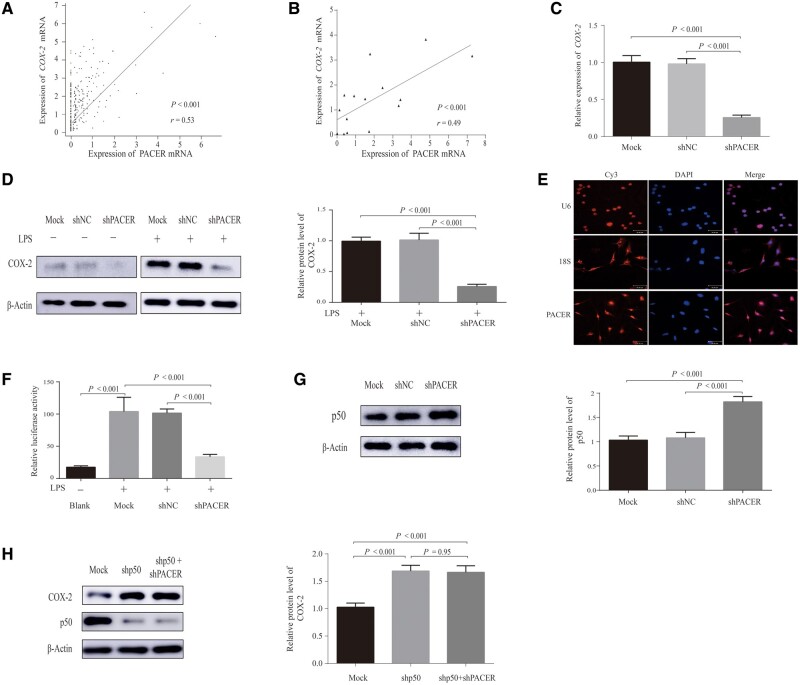
PACER mainly acts on the promoter region and activates *COX-2* transcription via the NF-κB pathway in CRC cells. (A) The correlation analysis of mRNA expression was conducted for PACER/*COX-2* in the TCGA databases (*P* < 0.001). (B) The correlation analysis of PACER/*COX-2* in 15 cancer-tissue samples (*P* < 0.001). (C) RT-qPCR assay was performed to measure the mRNA expression of *COX-2* in shPACER-transfected SW480 cells (*P* < 0.001). Data are shown as means ± SD (*n* = 3). (D) The SW480 cells were transfected with shPACER plasmid (shPACER group) or shRNA-negative control (shNC group), then stimulated with LPS for 8 h. The expression of COX-2 proteins was then quantified (*P* < 0.001). β-actin served as a loading control. Data are shown as means ± SD (*n* = 3). (E) The lncRNA fluorescence *in situ* hybridization assay revealed that U6 was mainly located in the nucleus and 18S was located in the cytoplasm as controls. The specific probes of PACER were hybridized into SW480 cells and the fluorescence was observed. (F) The luciferase activity of each group after stimulation with LPS for 8 h (*P* < 0.001). Blank, unstimulated cells. Data are shown as means ± SD (*n* = 3). (G) The relative protein expression of p50 in different groups (*P* < 0.001); β-actin served as control. (H) The relative protein expression of COX-2 in different groups. PACER, p50-associated cyclooxygenase-2 extragenic RNA; COX-2, cyclooxygenase-2; CRC, colorectal cancer; NC, negative control; LPS, lipopolysaccharide. –, non-treated; +, treated.

Our analyses of the TCGA database (*P* < 0.001) as well as CRC tumor samples (*n* = 15; *P* < 0.001) uncovered a positive relationship between the mRNA expression of PACER and that of *COX-2* (Figure 3A and B). RT-qPCR analysis revealed that knocking down PACER led to a significant reduction in *COX-2* expression (*P* < 0.001; Figure 3C), suggesting that PACER might enhance *COX-2* mRNA expression. We then investigated COX-2 protein levels using Western blotting (Figure 3D). This analysis revealed that, while COX-2 protein was modestly detectable in the absence of external stimulation, it was almost undetectable in upon PACER knock-down. Upon *COX-2* stimulation using LPS, it was observed that, relative to the control group, COX-2 protein levels were much lower following PACER knock-down (*P* < 0.001).

To explore the mechanism through which PACER regulates *COX-2*, we first performed lncRNA FISH using PACER-specific lncRNA probes (Figure 3E). As controls, U6 and 18S RNA mainly localized in the nucleus and cytosol, respectively. In contrast, *in situ* hybridization analysis revealed that PACER largely localizes in the nucleus, suggesting that this lncRNA may regulate nuclear processes including transcription [33]. Next, we performed a luciferase assay by transfecting a plasmid bearing shPACER and the *COX-2* promoter luciferase-reporter plasmid (XP2-h*COX-2*-LUC) into SW480 cells. We then stimulated the cells with LPS to activate *COX-2* production and analysed the luciferase activity (all *P* < 0.001; Figure 3F). After LPS stimulation, the control-group cells had significantly higher relative luciferase activity than the blank group (unstimulated cells). The result revealed suppressed reporter activity in the presence of shPACER relative to the mock group, suggesting that PACER acts on the *COX-2* promoter region to drive *COX-2* transcription. Previous research had reported that PACER promoted *COX-2* expression by directly binding and suppressing the NF-κB subunit p50, thereby enhancing p65-positive modulation on the *COX-2* promoter [28]. We then used Western blotting to quantify the protein level of p50 (Figure 3G). The result showed that p50 protein increased when PACER was knocked down (*P* < 0.001). This analysis revealed that PACER could decrease p50 protein in CRC cells. As shown in Figure 3H, the expression level of COX-2 increased significantly after p50 knock-down (*P* < 0.001). Subsequently, PACER was knocked down on this basis and the level of COX-2 did not decrease concomitantly. This demonstrated that PACER can only regulate COX-2 expression in the case of p50 presence. Our data suggested that PACER may play the regulatory role of COX-2 through interacting with the p50, which is the important repressive subunit of the NF-κB pathway.

Taken together, these findings suggest that PACER might act on the promoter region of the *COX-2* gene to activate *COX-2* expression via the NF-κB pathway in CRC cells.

### Loss of PACER suppresses CRC development *in vivo*

**Figure 4.**
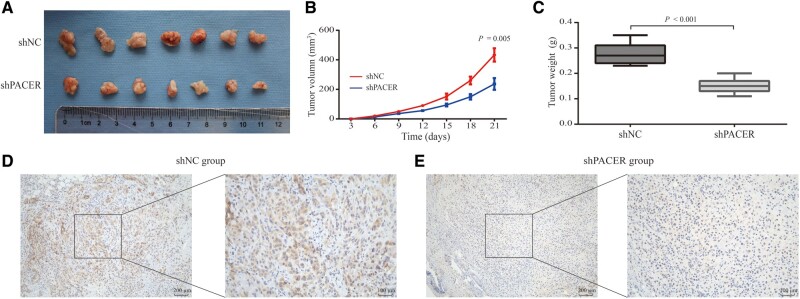
Knock-down of PACER repressed CRC progression *in vivo*. (A) SW480 cells with stable transfection of shPACER were subcutaneously inoculated into nude mice for 21 days, after which the xenografted tumors were resected and weighed. (B) The tumor volumes in the two groups (*P* = 0.005). Data are shown as means ± SD (*n* = 3). (C) Weight of the excised tumors in the two groups (*P* < 0.001). Data are shown as means ± SD (*n* = 7). (D) The images of COX-2 protein in tumor tissues excised from the shNC group. 100× magnification (left), 200× magnification (right). (E) The immunohistochemical-staining images of COX-2 protein in tumor tissues excised from the shPACER group. 100× magnification (left), 200× magnification (right). PACER, p50-associated cyclooxygenase-2 extragenic RNA; COX-2, cyclooxygenase-2; CRC, colorectal cancer; NC, negative control.

To clarify whether the downregulation of PACER could inhibit the progression of CRC *in vivo*, stable PACER-knock-down SW480 cells were subcutaneously inoculated into nude mice (Figure 4A). The weight and volume of tumors were measured as the primary indices. As shown in Figure 4B, the volumes of tumors were smaller in the PACER-knock-down group than in the control group (*P* = 0.005). At 21 days after injection, the tumors were excised and weighed. The weight of tumors was significantly lighter in the PACER-knock-down group (*P* < 0.001; Figure 4C). Taken together, these data sets demonstrated that downregulation of PACER expression also retarded the growth of CRC tumors *in vivo*. Immunohistochemical-staining results demonstrated that COX-2 was more highly expressed in the control group (Figure 4D) than in the PACER-knock-down group (Figure 4E). Collectively, these findings confirm that knock-down of PACER repressed CRC progression *in vivo*, and this was associated with reduced COX-2 protein expression.

### PACER knock-down inhibits CRC proliferation and invasion in CRC cells suppressing PGE2 expression

**Figure 5.**
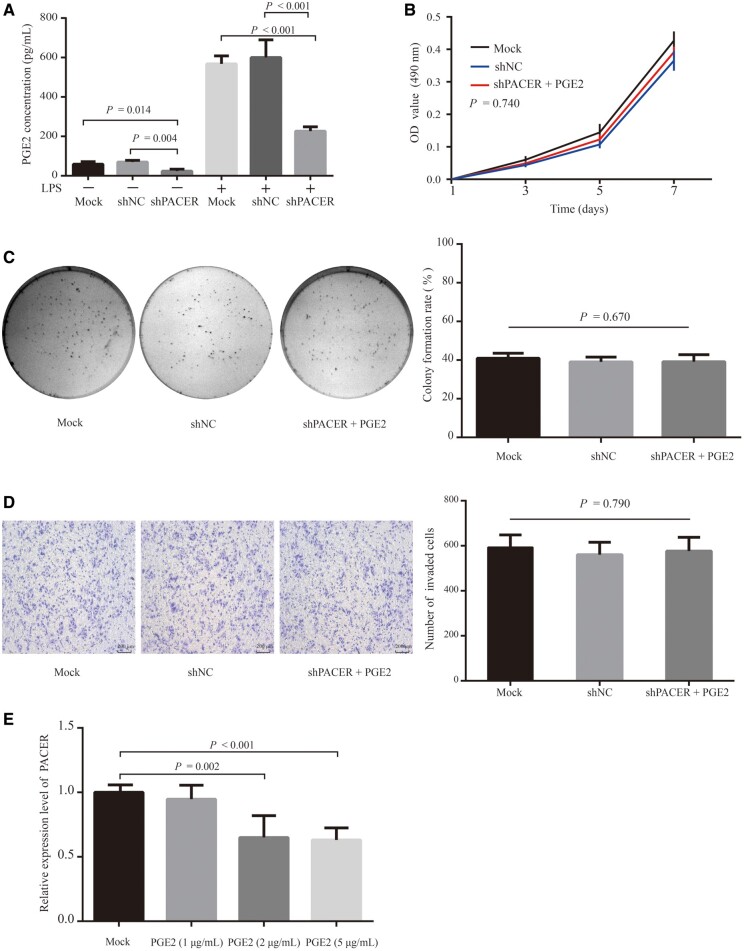
PACER regulated the production of PGE2 in CRC cells, and co-treated PGE2 increased the proliferation and invasion of PACER-knock-down cells. (A) The concentration of PGE2 after stimulation with LPS as determined by ELISA. Data are shown as means ± SD (*n* = 3). (B) The proliferation ability of SW480 cells after treatment with PGE2 as measured by MTT assay. There was no significant difference between the groups (*P* = 0.740). Data are shown as means ± SD (*n* = 3). (C) Colony-formation rate in each group. There was no significant difference between the groups (*P* = 0.670). Data are shown as means ± SD (*n* = 3). Crystal violet staining (200× magnification). (D) The invasion ability of SW480 cells in the shPACER group after treatment with PGE2 as determined by transwell assay. There was no significant difference between the groups (*P* = 0.790). Data are shown as means ± SD (*n* = 3). (E) The mRNA expression of PACER in SW480 cells after treatment with PGE2 for 6 h as measured by RT-qPCR assay. Data are shown as means ± SD (*n* = 3). PACER, p50-associated cyclooxygenase-2 extragenic RNA; CRC, colorectal cancer; NC, negative control; LPS, lipopolysaccharide.

It is well established that LPS stimulation activates NF-κB signaling and PGE2 production [34]. We next stimulated CRC cells with LPS and used ELISA to measure the levels of PGE2 secreted into cell-culture media following. This analysis revealed that PGE2 levels were strongly enhanced by LPS treatment (Figure 5A), relative to unstimulated cells. More importantly, knock-down of PACER significantly reduced PGE2 levels in the shPACER group, whether with LPS treatment or without LPS treatment.

We co-treated PGE2 and PACER-knock-down cells and performed MTT and colony-formation assays to monitor cell proliferation. This analysis revealed that co-treatment with PGE2 restored cell proliferation and there was no significant difference among the three groups (*P* = 0.740; Figure 5B). Similar observations were made for the colony-formation and transwell-migration assays (*P* = 0.670, Figure 5C;*P* = 0.790, Figure 5D). Taken together, these results indicate that the introduction of PGE2 counters the inhibition of CRC proliferation, invasion, and migration induced by PACER knocked down.

We treated SW480 cells with varying concentrations of PGE2 (1, 2, or 5 μg/mL) and measured the expression of PACER by RT-qPCR. This analysis revealed that treatment of the CRC cells with 2 μg/mL (*P* = 0.002) or 5 μg/mL (*P* < 0.001) significantly reduced PACER levels relative to the mock group (Figure 5E), suggesting that, through a negative feedback loop, high PGE2 levels act back to downregulate *COX-2* through inhibiting PACER-expression levels.

## Discussion

*COX-2* is known to promote tumorigenesis through prostanoid biosynthesis [35]. PGE2 is one of the prostanoid products that is strongly associated with the proliferation, migration, and invasion of CRC cells [36, 37]. A previous report indicates that *COX-2* is highly expressed in human CRC and is frequently correlated with poor prognosis [38]. While it is established that *COX-2* expression is stimulated by cytokines and inflammatory triggers, the mechanism through which *COX-2* becomes upregulated in CRC is still continuously being explored. Professor Bitarte and his team indicated that miR-451 decreases in CRC and its downregulation induces the expression of the direct target gene macrophage migration inhibitory factor involved in the upregulation of *COX-2*. In turn, *COX-2* allowed Wnt activation, which is essential for cancer-stem-cell growth [39]. Another study from Li *et al*. revealed that YAP increases *COX-2* expression at the level of transcription by interacting with TEAD binding sites in the *COX-2* promoter, then YAP mediates drug resistance through *COX-2* and its related effectors [40].

Previous studies have identified several lncRNAs thought to indirectly modulate *COX-2*. It has been reported that the lncRNA CCHE1 is elevated in CRC tissues and is correlated with p-ERK1/2 signaling. This lncRNA has been shown to activate the ERK pathway and to promote cell proliferation via enhanced *COX-2* and cyclin D1 expression [41]. MIAT, another lncRNA, has been reported to act as a molecular sponge for the *COX-2* modulator, miR-216a-3p. This lncRNA can overturn the inhibitory effect of miR-216a-3p on *COX-2* and promote the activation and proliferation of human pancreatic stellate cells [42]. The lncRNA GAS5 is reported to promote the ubiquitin-mediated degradation of COX-2 [43].

PACER is a newly identified antisense lncRNA located on the *COX-2* promoter region and has been shown to increase *COX-2* expression in monocytes [28]. Mechanistically, PACER is thought to activate NF-κB signaling by sequestering its inhibitor, the p50 homologous dimer, and sequesters. This in turn recruits the NF-κB subunit p65 to the *COX-2* promoter and enhances *COX-2* transcription. A previous study that used RNA sequencing proposed that the lncRNA-PACER is highly associated with the expression of *COX-2* mRNA and that the PACER-*COX-2* axis regulates the stiffness of sclerotic cells [44]. It has also been reported that PACER is a modulator of cell fate in the hematopoietic system; PACER is known to regulate human macrophage differentiation in response to LPS stimulation, as well as malignant hematopoiesis by inducing abnormal *COX-2* expression [45]. However, as far as we know, relevant reports on the role of PACER in CRC are still lacking.

**Figure 6.**
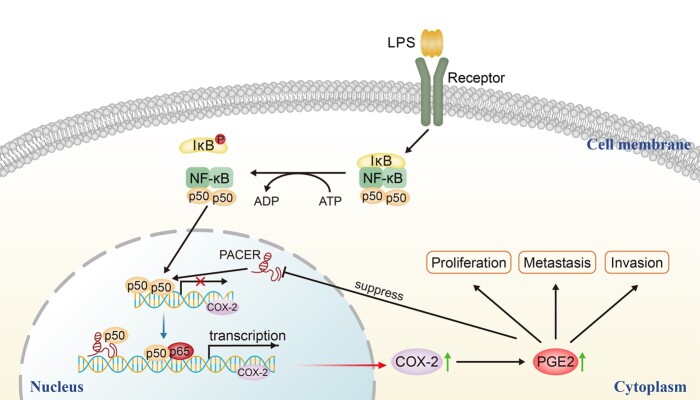
LPS, TNF-α, and other physiological or non-physiological stimuli regulate the phosphorylation and subsequent degradation of IκB, and the subsequent nuclear translocation of NF-κB. The homologous dimer of NF-κB1, which comprises two p50 subunits, is inactive for *COX-2* transcription because of the lack of an activated domain. PACER physically interacts and sequesters excessive p50, and transforms the homologous dimer p50/p50 into a p65/p50 heterodimer with transcription activity, thus activating the transcription of *COX-2*. Increased *COX-2* enhances PGE2 synthesis, which promotes the proliferation, invasion, and metastasis of CRC cells. In turn, the high expression of PGE2 decreases PACER expression and provides a negative feedback. PACER, p50-associated cyclooxygenase-2 extragenic RNA; COX-2, cyclooxygenase-2; CRC, colorectal cancer; LPS, lipopolysaccharide; NF-κB, nuclear factor kappa B.

In our study, we revealed that high expression of PACER may modify the initiation and progression of CRC. Our analysis demonstrated that PACER is highly expressed in CRC tissues and cell lines. Meanwhile, its expression correlates with increased *COX-2* expression in CRC. This is consistent with the previous report [46]. We speculated that the inhibition of CRC-cell proliferation upon PACER knock-down may be due to its effects on *COX-2* and its product PGE2. Our data indicated that addition of PGE2 into PACER-knock-down cells counters the negative effects of PACER knock-down on cell proliferation, migration, and invasion. Further analysis found that PACER exerts its biological effects by altering the expression of PGE2. Furthermore, it has been reported that *COX-2* is downregulated in the late stage of disease [35]. Previous studies have shown that the *COX-2* products PGE2 and PGJ2 participate in this process but the mechanism of action is unclear [47, 48]. We find that treatment of cells with PGE2 down-modulates PACER mRNA levels, suggesting the existence of a negative feedback in loop that may account for the reduced *COX-2* levels in the terminal stages of disease (Figure 6).

*COX-2* inhibits B-cells and natural killer T (NKT) cells, which in turn limits the power of immunosuppressors to combat tumor growth [49]. Through this mechanism, *COX-2* may promote tumorigenesis. Additionally, *COX-2*-PGE2 inhibits specific T-cell-induced cytotoxicity, promotes macrophage differentiation, increases infiltration by regulatory T-cells, and suppresses cytotoxic T-cell activity [50]. Taken together, this suggests that inhibition of PACER activity can be used as a means to downregulate *COX-2*, thus potentiating the capacity of immune cells to suppress tumor growth [51].

However, there are several limitations in our present study. First, the CCD-841 cell line is a relevant control cell line for studies of CRC, but it does not contain keratin. Second, we did not study the effects of PACER knock-down in low-PACER-expressing CRC-cell lines. Thus, it cannot be completely excluded that our results may be from non-specific effects of PACER knock-down. Third, we did not intensively investigate the mechanism involved when PGE2 down-modulates PACER. CREB is an important cAMP-responsive transcription factor. It has been found to be a constant stimulus of PGE2-induced abnormal cAMP-CREB signaling as well as the imbalance of inflammation [52]. we will further investigate whether CREB is involved in the regulation of PACER in future experiments.

In conclusion, our study has demonstrated the overexpression of PACER in CRC and its expression correlates with that of *COX-2*. We also found that PACER promotes proliferation, invasion, and metastasis of CRC cells. Additionally, we showed that PACER upregulates *COX-2* transcription and promotes PGE2 synthesis through the NF-κB-signaling pathway. This study uncovers a novel mechanism through which PACER may regulate *COX-2*, thereby promoting CRC-cell proliferation and invasion. Our findings indicate that PACER is a potential therapeutic target for the modulation of *COX-2* transcription and consequently the treatment of CRC.

## Authors’ contributions

P.S. and X.S.W. contributed to the study concept and design. P.S., J.C.Q., S.W., and M.Z. performed the experiments. Z.L., X.G., G.Y.W., and H.Y.W. provided the study materials and patients. P.S. and J.C.Q. collected the data and performed statistical analysis. P.S. drafted the manuscript. All authors reviewed and approved the final manuscript.

## Funding

This work was supported by National Natural Science Foundation of China [No.81572930], Beijing Science and Technology Plan [No.D171100002617004], and the Scientific Research Foundation of Graduate School of Harbin Medical University [YJSCX2017-63HYD].
